# Supplementary material for: A randomised controlled trial of raw honey for the healing of ulcers in leprosy in Nigeria
Source: PLoS Negl Trop Dis. 2025 Dec 31;19(12):e0013454. doi: 10.1371/journal.pntd.0013454 (PMC12774343; doi:10.1371/journal.pntd.0013454)
Supplement: S1 Text — Contains details of the information sheet that was handed out and explained to the participants to decide to voluntarily participate in the trial. (DOCX) [file pntd.0013454.s001.docx]

**Honey Experiment on LeProsy Ulcer (HELP): A Randomised Control Trial of Raw, Unadulterated African Honey for Ulcer Healing in Leprosy**

**Participant Information Sheet**

**Introduction**

We would like to invite you to take part in a research study. Joining the study is entirely up to you. Before you decide, you need to understand why the research is being done and what it would involve. One member of our team will go through this information sheet with you, and answer any questions you may have. Ask questions if anything you read is not clear or you would like more information. Please feel free to talk to others about the study if you wish. Take time to decide whether or not to take part.

**Who is organising and funding the study?**

The study is being organised by The Leprosy Mission Nigeria and RedAid Nigeria (Formerly GLRA Nigeria) in collaboration with the University of Birmingham, UK. The study is funded by the UK National Institute for Health Research.

**What is the purpose of the study?**

Leprosy ulcers are not caused by the leprosy germ but by loss of sensation leading to repetitive injury. Treatment consists of keeping the ulcer clean and fresh and also applying wet bandages regularly – dressing changes. You will currently have these dressing changes every 3 or 4 days.

The purpose of our study is to test a new method that may help the ulcer to heal faster. This treatment is done while you have your dressing changed. We will use honey to dress the wound for some of you while others will receive normal saline dressing on their wounds. The selection of participants for the honey or normal saline dressing will be done randomly. Honey is being used for wound dressing for centuries but this time, we want to do it as a registered trial.

At present, this treatment appears to be very safe, although we do not know if it works. This study only looks at ulcers on the feet or legs and not anywhere else.

**Why have I been asked to take part?**

You have been invited because you have ulcer in your foot.

**Do I have to take part?**

No. It is up to you to decide to take part or not. If you don’t want to take part, that’s ok. Your doctor will still care for you and your decision will not affect the quality of care you receive.

We will discuss the study together and give you a copy of this information sheet. If you agree to take part, we will then ask you to sign a consent form.

**What will happen to me if I take part?**

If you are willing to take part in this study, we will first ask you to sign a consent form which is your indication that you understand the study and agree to take part.

Then you will be evaluated, do an interview and physical examination of your ulcer in your foot. If you meet the study’s criteria, and you wish to participate, you will receive dressing with normal saline or with honey. The treatment will be chosen by chance by a computer so that half of the people in the trial get the normal saline (control group) and the other half get the honey (intervention group). It is really important that the two groups for this study have a similar mix of patients in them. Having a similar mix means that we know that if one group of participants does better than the other, it is very likely to be because of the treatment and not because there are differences in the types of patients in each group. You will have an equal chance of receiving either normal saline dressing or honey dressing. If you get enrolled in the study you will be asked to wear a pedometer on the ankle of non-affected limb. This is to monitor your movement during the hospital admission period from the first dressing change to discharge.

It is important that you realise that treatment is not always effective. If you agree to take part of this study, we will ask you to complete different questionnaires. You will be called for follow-up six months after randomisation for the trial.

**What will I have to do?**

You will be expected to be admitted in hospital during the treatment period. You have to answer the entire questions asked to you. This will help us to gather information about you and your progress during the study period.

**What information will be collected?**

Only simple information about you, your treatment for your ulcer and how it affects you will be collected. This will include your name, but you will only ever be viewed by your participant number. We will keep this information separate from your address.

We will also take photographs of your ulcer every time you have your dressing changed to see how well it is healing. These photographs will only ever be viewed by your participant number. We may also make video of your dressing change.

**What will happen to information collected about me?**

All information collected about you will be kept private. Only the study staff and authorities who check that the study is being carried out properly will be allowed to look at information about you. Data may be sent to other study staff at University of Birmingham but this will be anonymised. This means that any information about you which leaves the hospital/surgery/clinic will have your name and address removed so that you cannot be recognised.

Your doctor will send some details about you to the study team at university of Birmingham, who will store it securely. Your personal details will be kept in a different safe place to the other study information and will be kept for at least 10 years after study completion. All the data will be securely stored in safe place.

The collected data may also be used for future research, including impact activities following review and approval by an independent Research Ethics Committee and subject to your consent at the outset of this research project.

For further information, please refer to the University of Birmingham Research Privacy Notice which is available here: <https://www.birmingham.ac.uk/privacy/index.aspx> or by contacting the Information and Data Compliance Team at: [dataprotection@contacts.bham.ac.uk](mailto:dataprotection@contacts.bham.ac.uk).

**What if something goes wrong?**

If you have a concern about any aspect of this study, you should ask to speak to the researchers who will do their best to answer your questions. You can also contact Dr. Sunday Udo who is the principal investigator of this study for any queries. If you remain unhappy and wish to complain formally, you can do this by contacting Professor Richard Lilford, University of Birmingham UK, [r.j.lilford@bham.ac.uk](mailto:r.j.lilford@bham.ac.uk)

The study holds insurance policies which apply to this study. If you experience harm or injury as a result of taking part in this study, you may be eligible to claim compensation.

**Can I change my mind about taking part?**

Yes. You can withdraw from the study at any time. You just need to tell your doctor that you don’t want to be in the study anymore. Your doctor will still care for you.

You can withdraw from treatment but keep in contact with us to let us know your progress. Information collected may still be used.

This would not affect the care you receive. If the intervention proved effective, you will be eligible to receive it if you develop a new ulcer or if your ulcer has not healed or recur.

**What will happen to the results of this study?**

The study results will be published in a medical journal so that other doctors can learn from them. Your personal information will not be included in the study report and there is no way that you can be identified from it.

**Who has reviewed the study?**

All research involving human participants is looked at by an independent group of people, called a Research Ethics Committee, to protect your interests. This study has been reviewed and given favourable opinion by University of Birmingham’s Science, Technology, Engineering and Mathematics (STEM) ethics committee.

**Who should I contact if I want further information?**

Dr Sunday Udo, TLM Nigeria, Tel: +234 8090850600,

email: [sundayudoTLMN@gmail.com](mailto:sundayudoTLMN@gmail.com)

Dr Anthony Meka, RedAid Nigeria (Formerly GLRA Nigeria), Tel: +234 8037028796

Email: anthony.meka@redaidnigeria.org

Professor Richard Lilford, University of Birmingham, UK [r.j.lilford@bham.ac.uk](mailto:r.j.lilford@bham.ac.uk)

*****Thank you for taking time to read this information leaflet. If you think you will take part in the study please read and sign the consent form.*****
