# Supplementary material for: A randomised controlled trial of raw honey for the healing of ulcers in leprosy in Nigeria
Source: PLoS Negl Trop Dis. 2025 Dec 31;19(12):e0013454. doi: 10.1371/journal.pntd.0013454 (PMC12774343; doi:10.1371/journal.pntd.0013454)
Supplement: S2 Text — The detailed written informed consent document that was signed by all the participants before enrollment into the trial. (DOCX) [file pntd.0013454.s002.docx]

**CONSENT FORM**

**Title of Project:** **Honey Experiment on LeProsy Ulcer (HELP): A Randomised Control Trial of Raw, Unadulterated African Honey for Ulcer Healing in Leprosy**

**Name of Researcher(s):** Dr Sunday Udo, TLM Nigeria, Dr Anthony Meka, RedAid Nigeria (Formerly GLRA Nigeria), and Professor Richard Lilford, University of Birmingham, UK

A. I __________________________________________________________________ understand that doctors at the Leprosy Referral Hospital Chanchaga, St. Benedict Tuberculosis and Leprosy Relief Hospital, Ogoja, and at University of Birmingham are involved in research into alternative treatments methods for leprosy ulcers. This study will look at whether honey is more beneficial than normal saline dressing in healing foot ulcer or not. We are hoping to prove efficacy of new treatment method for healing leprosy ulcer.

B. The study has been explained to me.

C. I confirm that I am 18 years old or above.

D. I shall be randomly assigned to a normal saline dressing group or honey dressing group. There is equal chance of getting either normal saline dressing or honey dressing.

E. I agree to have photographs and videos taken during the ulcer dressing.

F. I agree that my collected data be used for further research in future*.

*Please note that participants may say ‘NO’ to this question and still take part in the study.

G. I can decide to leave the study at any time for any reason and will still receive other treatment from the hospital for my condition.

H. I understand that my name will not be revealed in any published material concerning this study. I understand that my notes will be treated with maximum confidentiality and will only be accessed by staff directly involved in the Study or the monitors of the Study.

I. I have received enough information about the study in a language I understand. I had the opportunity to discuss it and ask questions, and my questions have been answered to my satisfaction. I understand that participation is voluntary and that I am free to withdraw my consent at any time. I freely consent to participate in this research study and to allow treatment and tests to be performed on me as explained.

J. I understand that I can be requested anytime to terminate my participation in the trial if the need arises. I will be given full explanation of the reason and will still receive standard treatment.

K. I agree to take part in the study.

Printed Name & Signature (or finger print) Date

Name of Participant ________________________________

Signature/Finger Print ____________________________ _____/______/20____

Name of Witness ________________________________

Signature/ Finger Print ____________________________ _____/______/20____

Name of Researcher______________________________

Signature ________________________________ _____/______/20____
