# Supplementary material for: A randomised controlled trial of raw honey for the healing of ulcers in leprosy in Nigeria
Source: PLoS Negl Trop Dis. 2025 Dec 31;19(12):e0013454. doi: 10.1371/journal.pntd.0013454 (PMC12774343; doi:10.1371/journal.pntd.0013454)
Supplement: S3 Text — The final report prepared by the trial statisticians. (DOCX) [file pntd.0013454.s003.docx]

**Honey Experiment on LeProsy Ulcer (HELP): A Randomised Control Trial of Raw, Unadulterated African Honey for Ulcer Healing in Leprosy**

**Final Report**

**Prepared by:** Joshua Akinyemi

**Date:** 02 February 2025

**Table of Contents**

Contents

[**Trial Summary** 3](#_Toc189668386)

[**1.** **General Report Information** 5](#_Toc189668387)

[**2.** **Recruitment Details** 7](#_Toc189668388)

[**3. CONSORT Flow Diagram of Participants** 8](#_Toc189668389)

[**4. Data Completeness** 10](#_Toc189668390)

[**5. Participant Characteristics** 12](#_Toc189668391)

[**6. Adherence to Treatment Allocation** 15](#_Toc189668392)

[**7. Protocol Deviations** 16](#_Toc189668393)

[**8. Primary Outcome Measure** 17](#_Toc189668394)

[8.1 Subgroup analyses 20](#_Toc189668395)

[**9. Secondary outcomes** 22](#_Toc189668396)

[9.1 Sensitivity analysis: Inclusive analysis of outcomes measured at 6 months post randomisation 26](#_Toc189668397)

[**11. Analysis of Activity measurement** 27](#_Toc189668398)

[**12. Safety** 28](#_Toc189668399)

**List of Tables**

[Table 1: Reasons for participant exclusion 11](#_Toc191489437)

[Table 2: Participant trial exit (attrition) before discharge from hospital or 84 days after randomisation (whichever is earlier) 11](#_Toc191489438)

[Table 3: Form return rates for Dressing Change Form 12](#_Toc191489439)

[Table 4: Participant baseline characteristics 13](#_Toc191489440)

[Table 5: Treatment adherence 16](#_Toc191489441)

[Table 6: Protocol deviations 17](#_Toc191489442)

[Table 7: Analysis of primary outcome measure – Mean difference in wound area 19](#_Toc191489443)

[Table 8: Analysis of primary outcome measure - Time to complete re-epithelisation 19](#_Toc191489444)

[Table 9: Test of PH assumption 20](#_Toc191489445)

[Table 10: Subgroup analysis – Mean difference in wound area 21](#_Toc191489446)

[Table 11: Subgroup analysis - Time to complete re-epithelisation 22](#_Toc191489447)

[Table 12: Analysis of binary secondary outcomes 24](#_Toc191489448)

[Table 13: Summary of anatomical changes at 6 months post-randomisation 25](#_Toc191489449)

[Table 14: Analysis of continuous secondary outcomes 26](#_Toc191489450)

[Table 15: Sensitivity Analysis of binary secondary outcomes 27](#_Toc191489451)

[Table 16: Analyses of activity measurement (step count) at 7, 14, and 42 days post randomisation 28](#_Toc191489452)

**List of Figures**

[Figure 1: Numbers screened and randomised into HELP trial per month 9](#_Toc189670272)

[Figure 2: CONSORT flow diagram 10](#_Toc189670273)

[Figure 3: Plot of ulcer size over time 19](#_Toc189670274)

# **Trial Summary**

| **TABLE Trial** | |
| --- | --- |
| **Objectives** | To evaluate the healing properties of raw, undiluted African honey in comparison with normal saline dressing of leprosy ulcers. |
| **Trial design** | Single centre, prospective, single blinded, parallel group, blocked stratified 1:1 individually randomised controlled trial.  Figure 1 provides the trial schema. |
| **Total Number of Participants** | 130 |
| **Sample size assumptions** | We expect about 70% of ulcers to heal within 84 days with standard care (according to a recent study of neuropathic ulcers, over half due to leprosy). Assuming that the intervention will increase this proportion to 90% and hazards are constant and proportional (so that the hazard ratio is 1.91 for discharge), for a two-sided test of the hazard ratio with a type I error of 5% and statistical power of 80% and a 1:1 allocation ratio, 47 individuals are required in each group. With 130 participants, this allows for a drop out rate of up to 40% to achieve power of 80%. At the most pessimistic sample size of 90, with a drop out rate of 40%, the minimum detectable effect size (i.e. the effect size that achieves an 80% power with 33 patients per arm) is a hazard ratio of 2.15, or 92.5% of patients in the treatment group being discharged by end of the trial period. At the most optimistic sample size of 130 with no drop out, our minimum detectable effect size is a hazard ratio of 1.74, or 87.3% of patients being discharged in the treatment group. All calculations were based on a log-rank test |
| **Eligibility criteria** | The inclusion criteria are as follows:  1. Patients with a chronic foot ulcer of at least 6 weeks duration due to leprosy neuropathy.  2. ≥18 years of age.  3. Ulcer surface area > 2cm^2^  4. Ulcer is clean, dry, and free from infection.  5. Patient can provide informed consent.  The exclusion criteria are as follows:  1. Any significant medical condition, laboratory abnormality, or psychiatric illness that would prevent the participants from participating in the study  2. Ulcer with surface area <2cm^2^  3. Patient requires skin graft.  4. Any condition that confounds the ability to interpret data from the study (i.e., HIV, chronic Hep B, chronic Hep C or TB patients under active treatment).  5. Any wound that has clinical microbial infections.  6. Diabetes or Diabetic ulcer  7. A patient who has returned to the hospital having previously taken part in the trial. |
| **Interventions** | Raw, unadulterated honey applications at the time of dressing changes to ulcers twice per week. Controls get dressing change with normal saline |
| **Primary outcomes measure** | 1. Rate of healing based on one observation per week (cm^2^ per unit time). 2. Time to complete re-epithelisation (measured up to 84 days). |
| **Secondary outcome measures** | 1. Long-term (6 month) end-points will be: 2. Recurrence of treated ulcer; 3. Appearance of a new ulcer; 4. Anatomical changes in the limb;   Long-term endpoints will be measured at the time of follow up at 6 months from randomisation.  1. Days hospitalised prior to discharge and the total number of days hospitalised (to include any readmission related to leprosy-ulcers) by 6 months.  2. Number of visits to any healthcare facility from discharge to the end of follow-up at 6 months. |
| **Funder** | UK National Institute for Health Research (NIHR) Research and Innovation for Global Health Transformation (RIGHT) Programme |

1. **General Report Information**

| Protocol version number: | 0.8 |
| --- | --- |
| Date trial opened to recruitment: | 15 MAR 2022 |
| Date first participant was randomised: | 22 MAR 2022 |
| Date the dataset was frozen for analysis: | 13 DEC 2024 |
| Number of participants randomised on date the dataset was frozen for analysis: | 130 |
| % Target Recruitment  (Number of participants randomised / Total target sample size) | 100% (130/130) |
| Total randomised  Dressing changes with normal saline  Dressing changes with Honey | 130  65  65 |

1. **Recruitment Details**

Information on screening and trial randomisation is shown **Figure 1.** 146 participants were screened and 130 randomised into the trial.

Figure 1: Numbers screened and randomised into HELP trial per month

# **3. CONSORT Flow Diagram of Participants**

**Figure 3** details the flow of participants through the trial.

Further details on the reasons for participant exclusions and participant drop-out are given in **Table 1** and Table 2 respectively.

Figure 2: CONSORT flow diagram

**146 screened/ assessed for eligibility**

**Excluded (n=16)**

- Not meeting inclusion or meeting exclusion criteria (n=16)

**130 randomised**

**Honey: n=65**

**Withdrawn (n=3)**

**Lost to FU (n=0)**

**Died (n=0)**

**Normal saline: n=65**

**Withdrawn (n=7)**

**Lost to FU (n=0)**

**Died (n=0)**

**Lost to FU (n=9)**

**Lost to FU (n=10)**

**Died (n=1)**

**Excluded for protocol deviation=8**

**Died (n=2)**

**Excluded due to protocol deviation=7**

**ITT Analysis at 84 days (n=65)**

Note: *All participants were included and censored if needed at 84 days or their withdrawal date*

**ITT Analysis at 84 days (n=65)**

Note: *All participants were included and censored if needed at 84 days or their withdrawal date as*

**ITT Analysed at 6 months follow-up (n=49)**

*Implemented as sensitivity analysis including all participants was conducted.*

**ITT Analysed at 6 months follow-up (n=52)**

*Implemented as sensitivity analysis including all participants was conducted.*

**Analysed at 6 months follow-up (n=43)**

Note: *Excluded because their assessment was outside the window of 6 months ±1 month from randomisation.*

**Analysed at 6 months follow-up (n=40)**

Note: *Excluded because their assessment was outside the window of 6 months ±1 month from randomisation.*

Table 1: Reasons for participant exclusion

| **Reasons*** | **n(%)** |
| --- | --- |
| **Did not meet inclusion criteria** | **16 (11.0%)** |
| Patient didn’t have a chronic foot ulcer | 15 (10.3) |
| Patient did not have a chronic foot ulcer of at least 6 weeks duration due to leprosy neuropathy? | 5(3.5) |
| Patient < 18 years old | 1 (0.7) |
| Ulcer surface area <2 cm^2^ | 6 (4.1) |
| Ulcer is not clean, dry and free from clinical infection | 6 (4.1) |
| Patient is not able to understand and willing to voluntarily sign an informed consent document prior to any study related assessments being conducted | 1 (0.7) |
| **Did not want to participate in trial** | **0 (0.0)** |

***** *Reasons are not mutually exclusive.*

Table 2: Participant trial exit (attrition) before discharge from hospital or 84 days after randomisation (whichever is earlier)

|  | **n(%)** |
| --- | --- |
| **Number withdrawn** | **10 (7.7%)** |
| **Reasons** |  |
| 1. Relocating from Minna with family 2. Patient requested for discharge against medical advice to attend to issues in hometown 3. Participant wish to travel to reunite with family 4. The participant requested to be discharged so they can travel to attend to family/personal issues 5. The participant was recalled home by the parents, which was against the medical advice 6. Patient wish to go home to attend to family issues 7. Patient wish to return to community for schooling 8. Patient wishes to return to the community | 1  1  1  1  1  3  1  1 |
| **Number lost to follow-up** | **19 (14.6%)** |
| **Number of deaths** | **3 (2.3%)** |

# **4. Data Completeness**

**Table**  3 showed the return rates for key trial forms, for all randomised patients.

Table 3: Form return rates for Dressing Change Form

|  | **Dressing changes with Normal saline** | | | **Dressing changes with Honey** | | |
| --- | --- | --- | --- | --- | --- | --- |
| **Time-point ^¥^** | **No of Forms Expected ^a^** | **No of Forms Received** | **% Return** | **No of Forms Expected ^a^** | **No of Forms Received** | **% Return** |
| 1a week | 65 | 65 | 100% | 65 | 65 | 100% |
| 1b week | 65 | 65 | 100% | 65 | 65 | 100% |
| 2a week | 65 | 65 | 100% | 65 | 64 | 98% |
| 2b week | 64 | 64 | 100% | 65 | 65 | 100% |
| 3a week | 64 | 61 | 95% | 65 | 64 | 98% |
| 3b week | 64 | 58 | 91% | 65 | 65 | 100% |
| 4a week | 56 | 56 | 100% | 63 | 63 | 100% |
| 4b week | 56 | 55 | 98% | 63 | 63 | 100% |
| 5a week | 56 | 55 | 98% | 61 | 61 | 100% |
| 5b week | 56 | 55 | 98% | 61 | 61 | 100% |
| 6a week | 56 | 55 | 98% | 58 | 58 | 100% |
| 6b week | 56 | 55 | 98% | 58 | 57 | 98% |
| 7a week | 55 | 55 | 100% | 54 | 53 | 98% |
| 7b week | 55 | 53 | 96% | 54 | 54 | 100% |
| 8a week | 50 | 50 | 100% | 54 | 53 | 98% |
| 8b week | 50 | 51 | 102% | 50 | 49 | 98% |
| 9a week | 49 | 49 | 100% | 50 | 47 | 94% |
| 9b week | 49 | 48 | 98% | 46 | 45 | 98% |
| 10a week | 48 | 45 | 94% | 46 | 44 | 96% |
| 10b week | 48 | 45 | 94% | 46 | 41 | 89% |
| 11a week | 46 | 45 | 98% | 43 | 40 | 93% |
| 11b week | 46 | 43 | 93% | 40 | 38 | 95% |
| 12a week | 41 | 41 | 100% | 40 | 37 | 93% |
| 12b week | 41 | 41 | 100% | 40 | 37 | 93% |

# **5. Participant Characteristics**

The characteristics of the participants in the trial are summarised in **Table 4** .

Table 4: Participant baseline characteristics

|  |  | **Dressing changes with Normal saline**  **(n=65)** | **Dressing changes with Honey**  **(n=65)** | **Total**  **(n=130)** |
| --- | --- | --- | --- | --- |
| **Variables used in covariate adjustment** | |  |  |  |
| Trial ulcer Area^¥^ (cm^2^) - | n | 65 | 65 | 130 |
|  | Mean (SD) | 8.7 (8.3) | 9.6 (11.1)) | 9.1 (9.7) |
|  | Range | 2, 49 | 2, 63 | 2, 63 |
|  | Missing | 0 | 0 | 0 |
| Age at randomisation (years)^Ɨ^ | n | 65 | 65 | 130 |
|  | Mean (SD) | 48.3 (16.1) | 52.7 (13.8) | 50.5 (15.1) |
|  | Range | 18, 82 | 22, 82 | 18, 82 |
|  | Missing | 0 | 0 | 0 |
| **Participant demographics** |  |  |  |  |
| Gender, n (%) | Male | 42 (64.6) | 43 (66.2) | 85 (65.4) |
|  | Female | 23 (35.6) | 22 (33.9) | 45 (34.6) |
|  | Other | - | - | - |
|  | Missing | 0 | 0 | 0 |
| Highest level of education | Never joined formal school | 37 (56.9) | 40 (61.5) | 77 (59.2) |
|  | Primary level | 17 (26.2) | 16 (24.6) | 33 (25.4) |
|  | Secondary level | 6 (9.2) | 4 (6.2) | 10 (7.7) |
|  | Higher secondary level | 4 (6.2) | 5 (7.7) | 9 (6.9) |
|  | University level | 1 (1.5) | 0 (0.0) | 1 (0.8) |
|  | Missing | 0 | 0 | 0 |
| **Clinical information** |  |  |  |  |
| Height in cm | n | 65 | 65 | 130 |
|  | Mean (SD) | 162.8 (8.9) | 162.9 (8.2) | 162.8 (8.5) |
|  | Range | 148, 191 | 148, 188 | 148, 191 |
|  | Missing | 0 | 0 | 0 |
| Weight in kg | n | 65 | 65 | 130 |
|  | Mean (SD) | 55.4 (11.5) | 55.8 (12.4) | 55.6 (11.9) |
|  | Range | 35, 95 | 30, 90 | 30, 95 |
|  | Missing | 0 | 0 | 0 |
| BMI | n | 65 | 65 | 130 |
|  | Mean (SD) | 20.9 (4.3) | 21.0 (4.2) | 21.0 (4.2) |
|  | Range | 14.5, 37.6 | 12.5, 32.3 | 12.5, 37.6 |
|  | Missing | 0 | 0 | 0 |
| **Leprosy details** |  |  |  |  |
| Number of years since leprosy diagnosis | n | 65 | 65 | 130 |
|  | Mean (SD) | 18.8 (15.5) | 23.8 (16.6) | 21.3 (16.2) |
|  | Range | 1, 62 | 1, 63 | 1, 63 |
| Oral antibiotic (multi-drug therapy) treatment for leprosy | Ongoing | 10 (15.4) | 2 (3.1) | 12 (9.2) |
|  | Completed | 55 (84.6) | 63 (96.9) | 118 (90.8) |
|  | Unknown |  |  |  |
|  | Missing | 0 (0.0) | 0 (0.0) | 0 (0.0) |
| Diseases other than leprosy^*^ | Yes | 0 (0.0) | 0 (0.0) | 0 (0.0) |
|  | No | 64 (98.5) | 65 (100.0) | 129(99.2) |
|  | Missing | 1 (1.5) | 0 (0.0) | 1 (0.8) |
| **VMT/ST** |  |  |  |  |
| VMT/ST | Normal | 7 (10.8) | 3 (4.6) | 10 (7.7) |
|  | Impaired | 58 (89.2) | 62 (95.4) | 120 (92.3) |
|  | Missing | 0 (0.0) | 0 (0.0) | 0 (0.0) |
| Any nerve enlarged in leg | Left | 4 (6.9) | 1 (1.6) | 5 (4.2) |
|  | Right | 1 (1.7) | 1 (1.6) | 2 (1.7) |
|  | Bilateral | 11 (19.0) | 17 (27.9) | 28 (23.5) |
|  | Nil | 42 (72.4) | 42 (68.9) | 84 (70.6) |
|  | Missing | 0 (0.0) | 0 (0.0) | 0 (0.0) |
| Any loss of sensation in foot | Left | 6 (10.3) | 8 (12.9) | 14 (11.7) |
|  | Right | 5 (8.6) | 7 (11.3) | 12 (10.0) |
|  | Bilateral | 42 (72.4) | 39 (62.9) | 81 (67.5) |
|  | Nil | 5 (8.6) | 8 (12.9) | 13 (10.8) |
|  | Missing | 0 (0.0) | 0 (0.0) | 0 (0.0) |
| Any loss of motor function in foot | Left | 5 (8.6) | 5 (8.1) | 10 (8.3) |
|  | Right | 3 (5.2) | 2 (3.2) | 5 (4.2) |
|  | Bilateral | 13 (22.4) | 11 (17.7) | 24 (20.0) |
|  | Nil | 37 (63.8) | 44 (71.0) | 81 (67.5) |
|  | Missing | 0(0.0) | 0 (0.0) | 0 (0.0) |
| Any deformity in foot | Left | 13 (22.4) | 5 (8.1) | 18 (15.0) |
|  | Right | 5 (8.6) | 3 (4.8) | 8 (6.7) |
|  | Bilateral | 28 (48.3) | 37 (59.7) | 65 (54.2) |
|  | Nil | 12 (20.7) | 17 (27.4) | 29 (24.2) |
|  | Missing | 0 (0.0) | 0 (0.0) | 0 (0.0) |
| **Current Ulcer Information** |  |  |  |  |
| Total number of current ulcers, Left foot | n | 32 | 35 | 67 |
|  | Mean (SD) | 1.1 (0.8) | 1.2 (1.0) | 1.1 (0.9) |
|  | Range | 0, 3 | 0, 5 | 0, 5 |
|  | Missing | 0 | 0 | 0 |
| Total number of current ulcers, Right foot | n | 65 | 65 | 130 |
|  | Mean (SD) | 0.9 (1.0) | 0.9 (1.0) | 0.9 (0.9) |
|  | Range | 0, 5 | 0, 3 | 0, 5 |
|  | Missing | 0 | 0 | 0 |
| Location of the trial ulcer | Left forefoot | 19 (29.2) | 25 (38.5) | 44 (33.9) |
|  | Left midfoot | 26 (40.0) | 15 (23.1) | 41 (31.5) |
|  | Left hindfoot | 7 (10.8) | 9 (13.9) | 16 (12.3) |
|  | Right forefoot | 7 (10.8) | 5 (7.7) | 12 (9.2) |
|  | Right midfoot | 4 (6.2) | 6 (9.2) | 10 (7.7) |
|  | Right hindfoot | 2 (3.1) | 5 (7.7) | 7 (5.4) |
|  | Missing | 0 | 0 | 0 |
| Number of weeks trial ulcer unhealed | n | 65 | 65 | 130 |
|  | Mean (SD) | 95.2 (122.6) | 99.1 (124.9) | 97.1 (123.3) |
|  | Range | 6, 520 | 6, 520 | 6, 520 |
|  | Missing | 0 | 0 | 0 |
| Is the trial ulcer recurrent? | Yes | 20 (30.8) | 25 (38.5) | 44 (34.1) |
|  | No | 45 (69.2) | 40 (61.5) | 85 (65.9) |
|  | Missing | 0 | 0 | 0 |
| Time that the recurrent ulcer has been present in weeks | n |  |  |  |
|  | Mean (SD) | 95.5 (138.3) | 116.8 (156.8) | 107.6 (147.8) |
|  | Range | 4, 500 | 2, 500 | 2, 500 |
|  | Missing | 0 | 0 | 0 |
| **Measurements of the trial ulcer (for eligibility) ^ǂ^** | |  |  |  |
| Trial ulcer Max Length (cm) | n | 65 | 65 | 130 |
|  | Mean (SD) | 3.0 (1.6) | 3.1 (1.6) | 3.1 (1.6) |
|  | Range | 1.5, 9 | 1, 8 | 1, 9 |
|  | Missing | 0 | 0 | 0 |
| Trial ulcer Max Breadth (cm) | n | 65 | 65 | 130 |
|  | Mean (SD) | 2.5 (1.1) | 2.6 (1.3) | 2.6 (1.3) |
|  | Range | 1, 7 | 1, 9 | 1, 9 |
|  | Missing | 0 | 0 | 0 |
| Trial ulcer Area (cm^2^) | n | 65 | 65 | 130 |
|  | Mean (SD) | 8.7 (8.3) | 9.6 (11.1) | 9.1 (9.7) |
|  | Range | 2, 49 | 2, 63 | 2, 6 |
|  | Missing | 0 | 0 | 0 |

*Data are either mean (SD) or number (%)*

# **6. Adherence to Treatment Allocation**

**Table**  gives information on overall adherence to randomised treatment allocation. Adherence to allocated intervention was monitored by recording the type of treatment administered during dressing change(i.e. dressing changes with honey or dressing changes with normal saline). Adherent participants are those either allocated to the dressing changes with honey who receive honey in each dressing change, or those allocated to dressing changes with normal saline who do not receive honey in any dressing change. Non-adherence in the dressing changes will be defined as the failure to receive an allocated intervention.

Table 5: Treatment adherence

| **Time-point** |  | **Dressing changes with Normal saline**  **: (n=41)** | **Dressing changes with Honey**  **: (n=37)** |
| --- | --- | --- | --- |
| At 84 days or discharged (whichever is earlier) | Number of participants that always received randomised allocation | 41 (100.0%) | 37 (100.0%) |
|  | Number of participants that received alternate allocation at least once | 0 (0%) | 0 (0%) |
|  | Number of participants that received no intervention at least once | 1. (0%) | 0 (0%) |

#

# **7. Protocol Deviations**

The 6th month follow-up form was not completed within +/- 1 month for 15 participants

Table 6: Protocol deviations

| **Record ID** | **Deviation description** | **Dressing changes with Normal saline**  **: (n=65)** | **Dressing changes with Honey**  **: (n=65)** |
| --- | --- | --- | --- |
|  |  |  |  |
|  | 6-month follow-up assessment was after the time window | 7/49 (14.3%) | 8/52 (15.4%) |
|  |  |  |  |

# **8. Primary Outcome Measure**

There are two primary outcomes:

1) Rate of healing based on two observations per week (cm^2^ per unit time).

2) Time to complete re-epithelisation (observed at 84 days).

Figure 3: Plot of ulcer size (Log scale) over time


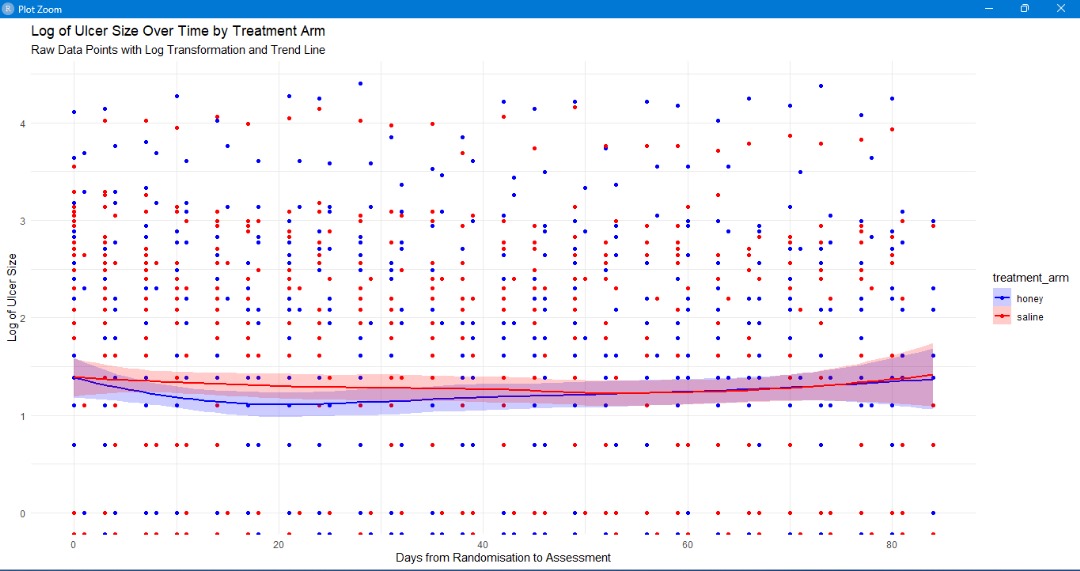


The estimates of treatment effect are shown in **Table 7** and **Table 8**

Table 7: Analysis of primary outcome measure – Mean difference in wound area

|  | Time point | Unadjusted^Ɨ^  Mean Difference^*^ (95% CI); p-value | Adjusted^ǂ^  Mean Difference^*^ (95% CI) ; p-value |
| --- | --- | --- | --- |
| **Ulcer Area measurements** | Day: 28 | 0.42 (-3.50, 4.34); 0.834 | -0.42 (-2.37, 1.54); 0.673 |
|  | Day: 56 | 1.37 (-2.63, 5.37); 0.499 | -0.58 (-2.71, 1.55); 0.588 |
|  | Day: 84 | 1.15 (-2.98, 5.29); 0.581 | 0.23 (-2.66, 2.21); 0.854 |

*^ǂ^ Mixed effects linear regression model adjusted for the baseline values of trial ulcer size and patient age. Baseline trial ulcer size and patient age was treated as continuous variables and considered as fixed effects in this adjustment.*

** Values of means differences<>0 indicate a larger average ulcer size per unit time in dressing changes with Honey vs Normal saline.*

Table 8: Analysis of primary outcome measure - Time to complete re-epithelisation

| **Time point** |  | **Dressing changes with Normal saline**  **(n=65)** | **Dressing changes with Honey**  **(n=65)** | **Unadjusted Hazard Ratio^*^**  **(95% CI)** | **Adjusted^ǂ^**  **Hazard Ratio^*^**  **(95% CI)** |
| --- | --- | --- | --- | --- | --- |
|  | Number of participants that withdrew before reached 84 days post randomisation | 5(7.7%) | 3 (4.6%) |  |  |
| At 84 days after randomisation | Number of Participants that had not reached complete re-epithelisation up to 84 days after randomisation | 44 (67.7%) | 43 (66.2%) | 1.17 (0.60, 2.27) | 1.26 (0.64, 2.47) |
|  | Number of Participants that had complete re-epithelisation up to 84 days after randomisation | 16 (24.6%) | 19 (29.2%) |  |  |

*^ǂ^ Cox proportional hazards adjusted for the baseline values of trial ulcer size and patient age. Trial ulcer size and patient age was treated as continuous variables and considered as fixed effects in the adjustment.*

*^*^ HR > 1 means – hazard of ‘healing’ is higher in dressing changes with Honey.*

**Test of proportional hazards assumption**

Table 9: Test of PH assumption

## 8.1 Subgroup analyses

Analysis was limited to the primary outcomes only, and a single pre-specified subgroup:

- Ulcer size at baseline above or equal to the median value at baseline vs ulcer size at baseline below the median value at baseline

The effect of the subgroup was examined by including a treatment group by subgroup interaction parameter in each primary outcome model. Estimates of mean difference in ulcer area at specified time points was stratified by the sub-group

Table 10: Subgroup analysis – Mean difference in wound area

| Time point | **Sub-group** | **Treatment and**  **subgroup interaction**  **term**  **P-value** | Unadjusted^Ɨ^  Mean Difference^*^ (95% CI) | **Treatment and**  **subgroup interaction**  **term**  **P-value** | Adjusted^ǂ^  Mean Difference^*^ (95% CI) ; p-value |
| --- | --- | --- | --- | --- | --- |
| Day: 28 | Baseline Ulcer Size >= Median=6.75 | 0.001 | 2.40 (-4.91, 9.71) | 0.192 | -0.09 (-3.82, 3.63) |
|  | Baseline Ulcer Size < Median=6.75 |  | -0.48 (-1.52, 0.57) |  | -0.93 (-1.97, 0.11) |
| Day: 56 | Baseline Ulcer Size >= Median=6.75 | 0.005 | 2.76 (-3.89, 9.41) | 0.422 | 0.24 (-3.24, 3.72) |
|  | Baseline Ulcer Size < Median=6.75 |  | -0.83 (-2.72, 1.06) |  | -1.99 (-3.96, -0.03) |
| Day: 84 | Baseline Ulcer Size >= Median=6.75 | 0.009 | 2.48 (-3.74, 8.69) | 0.870 | -0.002 (-3.77, 3.77) |
|  | Baseline Ulcer Size < Median=6.75 |  | -0.25 (-1.69, 1.20) |  | -1.18 (-2.71, 0.35) |

*^ǂ^ Mixed effects linear regression model adjusted for the baseline values of trial ulcer size and patient age. Baseline trial ulcer size and patient age was treated as continuous variables and considered as fixed effects in this adjustment.*

** * Values of means differences<>0 indicate a larger average ulcer size per unit time in dressing changes with Honey vs Normal saline*

Table 11: Subgroup analysis - Time to complete re-epithelisation

|  | **Unadjusted** | | **Adjusted^ǂ^** | |
| --- | --- | --- | --- | --- |
| **Sub-group** | **Treatment and**  **subgroup interaction**  **term**  **P-value** | **Hazard Ratio^*^**  **(95% CI)** | **Treatment and**  **subgroup interaction**  **term**  **P-value** | **Hazard Ratio^*^**  **(95% CI)** |
| Baseline Ulcer Size >= Median=6.75 | 0.169 | 3.68 (0.76, 17.69) | 0.824 | 3.89 (0.81, 18.79) |
| Baseline Ulcer Size < Median=6.75 |  | 0.77 (0.36, 1.67) |  | 0.81 (0.35, 1.83) |

*^*^ HR > 1 means – hazard of ‘healing’ is higher in dressing changes with Honey.*

*HR < 1 means – hazard of ‘healing’ is lower in dressing changes with Honey*

# **9. Secondary outcomes**

For the outcomes recorded on the 6-month post randomisation follow up questionnaire, their analysis includes participants who provided their responses within 1 month (+/-) of the 6-month post randomisation time point.

To examine the possible impact of the excluded data on the results, sensitivity analyses were performed on the 6-month follow-up outcome measures by including all participants.

The secondary outcomes are:

1. Recurrence of treated ulcer at 6 months from randomisation.
2. Appearance of a new ulcer at 6 months from randomisation.
3. Anatomical changes in the limb at 6 months from randomisation.
4. Total days hospitalised (to include any readmission related to leprosy-ulcers) by 6 months from randomisation.
5. Number of visits to any healthcare facility from discharge to the end of follow-up at 6 months from randomisation.

The results of these analyses are reported in Table 13 – Table 15.

Table 12: Analysis of binary secondary outcomes

|  | | **Dressing changes with normal saline (n=49)** | **Dressing changes with Honey (n=52)** | **Adjusted Relative Risk^1^**  **(95% CI)** | **Adjusted Risk Differnce^2^**  **(95% CI)** |
| --- | --- | --- | --- | --- | --- |
| **Recurrence of treated ulcer at 6 months from randomisation** | | | | | |
| Yes | | 5 (10.2%) | 7 (13.5%) | 1.34 (0.46, 3.92) | 0.04 (-0.10, 0.19) |
| No | | 37 (75.5%) | 37 (71.2%) |  |  |
| Missing | | 0 | 0 |  |  |
| Excluded from analysis | | 7 (14.3%) | 8 (15.4%) |  |  |
| **Appearance of a new ulcer at 6 months from randomisation** | | | | | |
| Yes | | 2 (4.1%) | 1 (1.9%) | 0.33 (0.04, 2.93) | - |
| No | | 40 (81.6%) | 42 (80.8%) |  |  |
| Missing | | 0 | 1 |  |  |
| Excluded from analysis | | 7 | 8 |  |  |
| **Anatomical changes in the limb at 6 months from randomisation** | | | | | |
| 6 months | Normal | 7 (14.3%) | 2 (3.9%) | 1.14 (0.98, 1.32)^3^ |  |
|  | Impaired | 35 (71.4%) | 42 (80.8%) |  |  |
|  | Missing | 0 | 0 |  |  |
|  | Excluded from analysis | 7 | 8 |  |  |

*1: Log-binomial regression model adjusted for the baseline values of trial ulcer size and participant age. Trial ulcer size and participant age were treated as continuous variables and considered as fixed effects in this adjustment. Adjusted RR > 1 means a higher rate of recurrent or new ulcers respectively was observed in the dressing changes with honey group..*

*2: Log-binomial regression model using the identity link function adjusted for the baseline values of trial ulcer size and participant age. Trial ulcer size and participant age were treated as continuous variables and considered as fixed effects in this adjustment. Adjusted RD > 0 means higher risk of recurrent or new ulcers respectively for the dressing changes with honey group.*

*3: Poisson regression model with robust standard errors adjusted for the baseline values of trial ulcer size and participant age. Trial ulcer size and participant age were treated as continuous variables and considered as fixed effects in this adjustment. Adjusted RR > 1 means a higher rate of normal limb was observed in the dressing changes with honey group.*

Table 13: Summary of anatomical changes at 6 months post-randomisation

|  | **Dressing changes with normal saline (n=35)** | **Dressing changes with**  **Honey (n=42)** |
| --- | --- | --- |
| Loss of sensation in foot |  |  |
| Left | 3 (7.5%) | 4 |
| Right | 1 (2.5%) | 3 (6.4%) |
| Bilateral | 27 (67.5%) | 33 (70.2%) |
| Nil | 4 (10.0%) | 2 (4.3%) |
| Loss of motor function in foot |  |  |
| Left | 6 (15.0%) | 3 (6.4%) |
| Right | 3 (7.5%) | 4 (8.5%) |
| Bilateral | 13 (32.5%) | 13 (27.7%) |
| Nil | 13 (32.5%) | 21 (44.7%) |
| Missing | 0 | 1 |
| Any deformity in foot |  |  |
| Left | 7 (17.5%) | 1 (2.1%) |
| Right | 3 (7.5%) | 2 (4.3%) |
| Bilateral | 14 (35.0%) | 21 (44.7%) |
| Nil | 11 (27.5%) | 18 (38.3%) |
| Palpable nerve in the leg |  |  |
| Left | 3 (7.5%) | 1 (2.1%) |
| Right | 2 (5.0%) | 2 (4.3%) |
| Bilateral | 10 (25.0%) | 10 (21.3%) |
| Nil | 20 (50.0%) | 29 (61.7%) |
| Changes in deformity of foot in comparison to baseline anatomy ^2^ | | |
| Yes | 0 (0.0) | 1 (2.1%) |
| No | 35 (87.5%) | 40 (85.1%) |
| Missing or excluded from analysis | 5 | 5 |

*1: Answers are not mutually exclusive.*

Table 14: Analysis of continuous secondary outcomes

|  | **Dressing changes with normal saline (N=65)** | **Dressing changes with Honey (N=65)** | **Adjusted Mean Difference^1^**  **(95% CI)** |
| --- | --- | --- | --- |
| **Days hospitalised prior to discharge** |  |  |  |
| n | 33 | 48 | 3.0 (-7.9, 13.9) |
| Mean (SD) | 54.6 (27.0) | 59.1 (21.2) |  |
| Min - Max | 10 - 84 | 17 - 84 |  |
| **Total days hospitalised during 6 months^2^** | | | |
| Excluded from analysis | 0 | 0 |  |
| n | 2 | 2 |  |
| Mean (SD) | 82.5 (89.8) | 107.0 (55.2) |  |
| Min - Max | 19 - 146 | 68 - 146 |  |

*1: Linear regression model adjusted for the baseline values of trial ulcer size and participant age. Trial ulcer size and participant age were treated as continuous variables and considered as fixed effects in this adjustment. Adjusted MD > 0 means higher total days hospitalised prior to discharge was observed in the dressing changes with honey group.*

*2: Out of 11 participants who were hospitalised within6 month follow-up, only 4 had complete data to estimate duration of hospitalisation*

## 9.1 Sensitivity analysis: Inclusive analysis of outcomes measured at 6 months post randomisation

For sensitivity analysis, we analysed all data recorded 6 months after randomization, regardless of whether they were recorded within 1 month (+/-) of the time point of 6 months after randomization. The following secondary outcomes were analysed:

- Recurrence of treated ulcer at 6 months from randomisation.
- Appearance of a new ulcer at 6 months from randomisation.
- Anatomical changes in the limb at 6 months from randomisation.

Table 15: Sensitivity Analysis of binary secondary outcomes

|  | | **Dressing changes with normal saline (n=49)** | **Dressing changes with Honey (n=52)** | **Adjusted Relative Risk^1^**  **(95% CI)** | **Adjusted Risk Differnce^2^**  **(95% CI)** |
| --- | --- | --- | --- | --- | --- |
| **Recurrence of treated ulcer at 6 months from randomisation** | | | | | |
| Yes | | 5 (10.2%) | 8 (15.4%) | 1.54 (0.54, 4.42) | 0.05 (-0.08, 0.18) |
| No | | 44 (89.8%) | 44 (84.6%) |  |  |
| Missing | | 0 | 0 |  |  |
| **Appearance of a new ulcer at 6 months from randomisation** | | | | | |
| Yes | | 3 (6.1%) | 3 (5.8%) | 0.98 (0.21, 4.56) | - |
| No | | 46 (93.9%) | 48 (92.3%) |  |  |
| Missing | | 0 | 0 |  |  |
| **Anatomical changes in the limb at 6 months from randomisation** | | | | | |
| 6 months | Normal | 9 (18.4%) | 5 (9.6%) | 1.09 (0.93, 1.28) ^3^ |  |
|  | Impaired | 40 (81.6%) | 47 (90.4%) |  |  |
|  | Missing | 0 | 0 |  |  |

# **11. Analysis of Activity measurement**

Table 16: Analyses of activity measurement (step count) at 7, 14, and 42 days post randomisation

| **Activity measurement** |  | **Dressing changes**  **with normal saline**  **(N=65)** | **Dressing changes with Honey**  **(N=65)** | **Mean Difference^1^**  **95% CI** |
| --- | --- | --- | --- | --- |
| Average daily steps measured  at 7 days post randomisation | N | 61 | 61 | 79.9  (-467.1, 626.9) |
|  | Mean (SD) | 1859.6 (1628.4) | 1779.7 (1415.5) |  |
|  | Min - Max | 0 – 6690.3 | 0 – 6263.4 |  |
|  | Missing | 4 | 4 |  |
| Average daily steps measured  at 14 days post randomisation | N | 56 | 61 | 39.0  (-540.4, 618.4) |
|  | Mean (SD) | 2039.5 (1678.8) | 2000 (1573.9) |  |
|  | Min - Max | 0 - 6993 | 0 - 7002 |  |
|  | Missing | 9 | 4 |  |
| Average daily steps measured  at 42 days post randomisation | N | 49 | 49 | 67.7  (-714.8, 850.3) |
|  | Mean (SD) | 2381.6 (1817.3) | 2313.9 (2076.8) |  |
|  | Min - Max | 0 – 6504.1 | 0 – 12388.9 |  |
|  | Missing | 16 | 16 |  |

*1: Mean difference is estimated using a t-test. MD>0 indicates higher mean daily steps in dressing changes with honey group.*

# **12. Safety**

Three deaths were reported: two in the Normal Saline arm and one in Honey arm. Two of the deaths occurred after the 24^th^ dressing change (from complications of herbal mixture and brief illness). The third death occurred during admission (due to sepsis on a non-trial ulcer)
