# Supplementary material for: A randomised controlled trial of raw honey for the healing of ulcers in leprosy in Nigeria
Source: PLoS Negl Trop Dis. 2025 Dec 31;19(12):e0013454. doi: 10.1371/journal.pntd.0013454 (PMC12774343; doi:10.1371/journal.pntd.0013454)
Supplement: S1 CONSORT Checklist — Checklist of items in accordance with the CONSORT guidelines (Adapted from https://journals.lww.com/asnjournals/Documents/CONSORT%202025.pdf). (PDF) [file pntd.0013454.s004.pdf]

CONSORT Checklist for Clinical Trials

Manuscript ID: PNTD-D-25-01353

Section/Topic	Checklist Item	Response
**Title**	Identified as a randomized trial in the title.	Yes
**Background**	Specific objectives or hypotheses clearly stated.	Yes
**Trial Design**	Trial design (such as parallel, factorial) including allocation ratio.	Yes
	Important changes to methods after trial commencement (such as eligibility criteria), with reasons.	Yes
**Participants**	Eligibility criteria for participants.	Yes
	Settings and locations where the data were collected.	Yes
**Interventions**	The interventions for each group with sufficient details to allow replication, including how and when they were actually administered.	Yes
**Outcomes**	Completely defined per-specified primary and secondary outcome measures, including how and when they were assessed.	Yes
	Any changes to trial outcomes after the trial commenced, with reasons.	Yes
**Sample Size**	How sample size was determined.	Yes
	Explanation of any interim analyses and stopping guidelines.	Yes
**Randomization**		
• **Sequence generation**	Method used to generate the random allocation sequence.	Yes
	Type of randomization; details of any restriction (such as blocking and block size).	Yes
• **Allocation concealment mechanism**	Mechanism used to implement the random allocation sequence (such as sequentially numbered containers), describing any steps taken to conceal the sequence until interventions were assigned.	Yes
• **Implementation**	Who generated the random allocation sequence, who enrolled participants, and who assigned participants to interventions.	Yes
**Blinding**	If done, who was blinded after assignment to interventions (for example, participants, care providers, those assessing outcomes).	Yes
	If relevant, description of the similarity of interventions.	Yes
**Statistical Methods**	Statistical methods used to compare groups for primary and secondary outcomes.	Yes
	Methods for additional analyses, such as subgroup analyses and adjusted analyses.	Yes
**Participant Flow (a diagram is strongly recommended)**	For each group, the numbers of participants who were randomly assigned, received intended treatment, and were analyzed for the primary outcome .	Yes
	For each group, losses and exclusions after randomization, together with reasons.	Yes
**Recruitment**	Dates defining the periods of recruitment and follow-up.	Yes
	Why the trial ended or was stopped.	Yes
**Baseline Data**	A table showing baseline demographic and clinical characteristics for each group.	Yes
**Numbers Analyzed**	For each group, number of participants (denominator) included in each analysis and whether the analysis was by original assigned groups.	Yes
**Outcomes and Estimation**	For each primary and secondary outcome, results for each group, and the estimated effect size and its precision (such as 95% confidence interval).	Yes
	For binary outcomes, presentation of both absolute and relative effect sizes.	Yes
**Ancillary Analyses**	Results of any other analyses performed, including subgroup analyses and adjusted analyses, distinguishing pre-specified from exploratory.	Yes
**Harms**	All important harms or unintended effects in each group.	Yes
**Data Sharing**	A data sharing statement is included.	Yes

Additional Details:

This checklist was dapted from https://journals.lww.com/asnjournals/Documents/CONSORT%202025.pdf
