# Supplementary material for: A randomised controlled trial of raw honey for the healing of ulcers in leprosy in Nigeria
Source: PLoS Negl Trop Dis. 2025 Dec 31;19(12):e0013454. doi: 10.1371/journal.pntd.0013454 (PMC12774343; doi:10.1371/journal.pntd.0013454)
Supplement: Attachment — Submitted filename: Response to reviewers PNTDs 09.12.25.docx [file pntd.0013454.s005.docx]

**PLOS Neglected Tropical Diseases submission (PNTD-D-25-01353): A randomised controlled trial of raw honey for the healing of ulcers in leprosy in Nigeria**

| **Comments** | Response |
| --- | --- |
| **Journal Requirements** |  |
| 1) Please ensure that the CRediT author contributions listed for every co-author are completed accurately and in full. At this stage, the following Authors/Authors require contributions: Paul Alumbugu Tsaku, Sunday Odihiri Udo, Pius Ogbu Sunday, Anthony Meka, Linda Chinonso Ugwu, Abiola Oladejo, Joshua Akinyemi, Akinyinka Omigbodun, Sopna Choudhury, Jo Sartori, Onaedo Ilozumba, Samuel Watson, and Richard Lilford. Please ensure that the full contributions of each author are acknowledged in the "Add/Edit/Remove Authors" section of our submission form. The list of CRediT author contributions may be found here: | We thank the editors for this reminder. We have now completed the full CRediT author contributions for all listed authors in the submission form, ensuring comprehensive and accurate acknowledgment of their roles (e.g., conceptualization, data curation, formal analysis, funding acquisition, investigation, methodology, project administration, supervision, validation, visualization, writing – original draft, and writing – review & editing). |
| 2) Please provide an Author Summary. This should appear in your manuscript between the Abstract (if applicable) and the Introduction, and should be 150-200 words long. The aim should be to make your findings accessible to a wide audience that includes both scientists and non-scientists. | We appreciate this requirement to enhance accessibility. An Author Summary (189 words) has been added to the revised manuscript between the Abstract and Introduction, summarizing the study's background, methods, key findings, and implications in plain language for a broad readership. |
| 3) We do not publish any copyright or trademark symbols that usually accompany proprietary names, eg ©,  ®, or TM  (e.g. next to drug or reagent names). Therefore please remove all instances of trademark/copyright symbols throughout the text, including: - TM on page: 10. | Thank you for noting this. All trademark symbols (including the TM on page 10) have been removed from the revised manuscript to comply with journal style. |
| 4) Please upload all main figures as separate Figure files in .tif or .eps format. For more information about how to convert and format your figure files please see our guidelines:  <https://journals.plos.org/plosntds/s/figures> | This has been addressed. All main figures have been exported and uploaded as separate high-resolution TIFF files, formatted according to the journal's guidelines. |
| 5) We have noticed that you referred to supplementary materials on page 22. However, there are no corresponding files uploaded to the submission. Please upload them as separate files with the item type 'Supporting Information'. | We apologize for the oversight. The supplementary materials (including the full analysis report, informed consent forms, CONSORT checklist, and study protocol reference) have now been uploaded as separate files under 'Supporting Information' in this resubmission. |
| **Methods** |  |
| Reviewer #1: Table 1: one of the characteristics is VMT/ST - this is the name of a test that includes many examinations, so what does normal and impaired VMT/ST mean in this context? The is a separate line for loss of sensation - so that is the ST part of 'VMT/ST. We then have 'loss of motor function' - what motor function is being tested in this context? is it only dorsi-flexion, or is it more than that? | We thank the reviewer for these insightful questions, which highlight important details on neuropathy assessment and practical implementation.  Regarding Table 1: Voluntary Muscle Testing (VMT) and Semmes-Weinstein Monofilament Testing (SWMT) represent a composite assessment of nerve function impairment (NFI) in leprosy, as per WHO guidelines. "Normal/impaired VMT/SWMT" provides a qualitative overall summary of peripheral nerve integrity at baseline, confirming the neuropathic etiology of the ulcer. The subsequent rows detail specific components: sensory loss (via SWMT thresholds on the affected foot, graded as impaired if >10g monofilament detection) and motor function (via VMT, scored 0-5 on the common peroneal nerve for ankle dorsiflexion and extensor hallucis longus strength; impaired if <4/5). These focused on the ulcer-bearing foot to ensure leprosy-related causality, with comprehensive NFI testing (including facial, ulnar, median, radial, and peroneal nerves) conducted for all participants. We have presented a summarized version of table in the revised manuscript following comments from another reviewer on the same table. We have relegated the full Table to Supplement Material S3. |
| The 84 days is based on a study that used total contact casts - this study did not use TCCs, so not comparable. | We acknowledge the lack of direct comparability with the total contact cast (TCC) study cited for the 84-day endpoint, as our trial did not employ TCCs. The 84-day duration was selected to standardize evaluation against a fixed timeframe for healing/re-epithelialization, allowing assessment of dressing efficacy while minimizing prolonged hospitalization in resource-limited settings. This aligns with ethical considerations and prior leprosy ulcer trials (e.g., Napit et al., 2020) and is not necessarily related to whether the treatment was the same or not. |
| As this is a research study, the honey was tested in a laboratory before being used, to ensure it was not contaminated with microbes. This would not be the case if used routinely. Do the authors have any advice or caution for using honey that has not been tested for contamination? | On honey contamination: Laboratory screening at NIPRD was a precautionary measure for trial integrity. Given honey's inherent low water activity and antimicrobial properties (e.g., hydrogen peroxide, methylglyoxal), which make significant microbial contamination rare in well-preserved raw honey. However, we state that “clinicians should weigh potential benefit against lack of definitive evidence” and we do not think we can go beyond the evidence presented in recommending whether or not any honey used in future should be tested. |
| Reviewer #3: The study's objective to test the hypothesis that raw honey improves the healing rates of leprosy-related ulcers compared to standard saline is clearly stated and testable. The dual-centre, single-blinded RCT design is appropriate, and the inclusion and exclusion criteria are well-defined.  Sample size justification is reasonable, although the power is reduced due to high attrition. Statistical analyses used in the study are suitable and well interpreted. Ethical procedures are well described with all the necessary approvals and informed consent in place. | We sincerely thank the reviewer for these positive comments and for recognizing the study's strengths in design, analysis, and ethics. |
| **Results** |  |
| Reviewer #3: The results are clearly presented and supported by appropriate tables. Figures, including the CONSORT flow diagram, are clear and informative. The primary outcome shows a modest but non-significant trend favouring honey. The secondary outcomes (recurrence, new ulcers) are presented clearly and support the main finding of no significant difference.  It could have been interesting to understand if any significant instructions on selfcare were provided post discharge, which had an influence on prognosis.  Figures are acceptable. | Thank you again for this remark. Our interest was for those healed on the trials to lead normal lives afterwards, hence, no significant precautionary words other than the regular leprosy self care was offered. |
| **Conclusions** |  |
| Reviewer #1: Honey is stated as a safe, affordable option in resource-limited settings. But we are not given the cost compared to normal saline. | Thank you for highlighting the practical implications of our findings regarding affordability in resource-limited settings. We have shortened our conclusions removing the conclusion that it is affordable because we did not study its costs and because we did not demonstrate superiority. |
| Reviewer #3: The conclusions are balanced and supported by the data. The authors appropriately highlight honey's potential safety and acceptability without overstating efficacy. Limitations and public health relevance are clearly discussed. | Thank you for this remark. |
| **Summary** **and** **general** **comments** |  |
| Reviewer #1: The study repeats the importance of pressure relief for healing wounds, rather than the wound cleaning material/solution. | Thank you for this perspective. We believe the manuscript appropriately contextualizes pressure relief (offloading) as a standardized co-intervention applied equally across arms, essential to isolate dressing effects and adhere to ethical standards (per WHO). This is briefly noted in the Methods (e.g., uniform discouragement of weight-bearing and step count monitoring to assess compliance) and Discussion (as part of multimodal care), without elaboration that overshadows the primary outcomes of healing rate and time to re-epithelialisation, which are analysed specifically for dressing effects (e.g., adjusted HRs in Tables 2–4). |
| Reviewer #2: Introduction: Para 1 - is it the pathogen itself or the immune response to that pathogen that causes nerve damage? Or both? | We have revised paragraph 1 of the Introduction for precision: "Nerve impairment results from both the direct effects of the causative organism, *Mycobacterium leprae* (*M. leprae*) complex in the peripheral nerves and skin, and the host's immune response to it, leading to inflammation and demyelination in peripheral nerves” |
| Para 2- in the list of other things people have tried I think it should be made clearer that the evidence for benefit for ost of these interventions is very minimal. | Thank you for this suggestion. We have amended paragraph 2 to state: “Additional methods to promote healing include leukocyte and platelet-rich fibrin (L-PRF) [6], biomaterials [10], and removable offloading devices, but none of these have been proven to be effective [11,12].” |
| Methods The formatting of the flow diagram is poor with words spilling out of the boxes etc. Please try and correct this. | The flow diagram has been formatted more appropriately. |
| Consent is not fully described - was this written consent? | We have indicated in the revised manuscript that it was a written informed consent. We have also included copies of the informed consent as supplementary materials. |
| Why were the specific study sites chosen? | The sites were selected for their specialization in leprosy care, established partnerships with the research team (The Leprosy Mission Nigeria and RedAid-Nigeria), and geographic diversity (north-central vs. south-south Nigeria) to enhance generalizability. We have added this rationale to the Study Setting subsection in Methods. |
| Please make clear in the methods that the trial was pre-registered (I can see this is given elsewhere but should also ideally be in the methods). | The trial registration (ISRCTN10093277, registered 22 December 2021) has been explicitly stated in the Trial Design subsection of Methods. The reference to the published protocol is also added to the section. |
| Please review the CONSORT checklist for RCTs and ensure that the paper is reported in line with this. Please submit a CONSORT checklist with the paper. | We have reviewed the manuscript against the CONSORT checklist. The CONSORT checklist is included in this resubmission. |
| Please describe the intervention in more details. For example does all the honey come from a single producer? Is there inter-batch variation? What fabric material was used for both control and intervention dressings? | Additional details in the study protocol have been published earlier. We have included the reference to the methods section. Both arms used sterile gauze dressings (non-adherent, cotton-based) applied post-cleaning. These align with the published protocol. |
| Is the approach taken to measuring ulcer size using photos and the software validated? Is there good reproducibility? And inter-observer agreement - it seems critical some evidence for this is provided as it serves as the key outcome measure. | The PictZar Digital Planimetry Software with PUSH tool is a validated method for wound area measurement, with high intra- and inter-observer reliability (ICC >0.95; e.g., Wunderlich & Perry, 2000) and compared favorably with other methods in a previous study (i.e., Wendelken et al, 2011: https://pubmed.ncbi.nlm.nih.gov/25879267/ and Ibraheem et al, 2023 https://pmc.ncbi.nlm.nih.gov/articles/PMC9863287/). |
| I am unclear if the primary outcome was the proportion healed at end of follow-up OR the time to healing? Was the protocol and the SAP published in advance? | The primary outcomes were (1) time to complete re-epithelialization (Cox model) and (2) healing rate (linear mixed-effects model), assessed up to 84 days post-randomization (not fixed follow-up proportion). The protocol and Statistical Analysis Plan (SAP) were pre-published (<https://www.ncbi.nlm.nih.gov/pubmed/38488451> and ISRCTN10093277), with no post-hoc changes. |
| What is the PRIMARY analysis - is it with or without adjustment. As currently written the stats plan is very difficult to decide what was specified a priori and what was post-hoc. Is the primary analysis ITT or PP? This isn’t stated. | As stated in the registered protocol (ISRCTN10093277, Section 12: Data analysis), we now state that “The primary analysis is the adjusted Cox proportional hazards model for time to complete healing (our key primary outcome), as this accounts for prognostic baseline factors (ulcer size and age) per standard practice in survival analyses for wound healing trials. All analyses are conducted on an intention-to-treat (ITT) basis, including all randomised participants with available outcome data (right-censored for non-healers at 84 days), consistent with CONSORT guidelines and the trial protocol. Unadjusted models and the linear mixed-effects analysis for healing rate are presented as supportive/secondary, with no post-hoc adjustments beyond the pre-specified multiple-testing correction (stepdown procedure on the two primary outcomes)”. |
| When someone had >1 ulcer how was the outcome ascertained? Time to healing of a specific ulcer? Of all ulcers? | Only the index ulcer (largest qualifying 2-20 cm² lesion) was assessed for the primary outcome; other ulcers received standard care but were not tracked for trial endpoints. This is stated earlier under ‘selection criteria’. |
| The trial was funded in the UK but there doesn’t appear to be any ethics approval in the UK? | In Nigeria, ethical approval was obtained from the National Health Research Ethics Committee (NHREC; ref: FHREC/2022/01/09/04-02-22). UK oversight was via institutional review at the University of Birmingham, confirming compliance with UK standards for international trials. This is mentioned in the manuscript. |
| Throughout there are averages given without measures of dispersion (i.e. the mean ulcer size was X but what was the SD, is a mean the best measure or a median?) I appreciate some of these are in tables but its good practice to include in the text too. | The SD have been included in the text. For ulcer size, the median and interquartile range has been provided. |
| Table 1 is very long. I suspect this could be summarised with more detailed information in a supplementary table. | Thank you for this practical suggestion. Table 1 has been condensed to key variables, leaving the other details as supplementary material. |
| In the results please avoid using language that suggests a positive difference when in fact there was no statistically significant differences found. for example ' " the slope of reduction was steeper in the honey-treated group, suggesting a more favourable healing trajectory. " O" With the unadjusted hazard ratio (HR) of 1.17 (95% CI: 0.60–2.27), honey had a 17% higher chance of healing compared to saline, but the confidence interval includes 1 (no effect), so this is not statistically significant." Please review all of the results thoroughly for such language. | We appreciate this important point on neutral reporting. This suggestion is noted and addressed in the manuscript. However, while remaining strictly neutral, we have mentioned the direction of the observed effect which aligns with the idea that hypothesis test are not decision rules. At the same time, and in recognition of the reviewers good point, we have toned down our conclusion to omit the phrase “the findings suggest that honey may be considered”, because, on reflection, we do not think the findings suggest that. Instead we suggest that anyone who is predisposed to use honey treatment should consider offering patients entry in rigorously conducted trials.  We have also altered the abstract in line with reviewers’ comments deleting ‘larger trials are needed to confirm efficacy’ and ‘provides crucial baseline data for future research on alternative therapies’. We now say that the trial provides ‘high quality data for inclusion in future systematic reviews’. |
| Figure 3 is incredibly hard to interpret. Please consider a clearer visualisation. | A clearer version of figure 3 has been included in the revised submission |
| Table 3 seems to be raw Stata output - is this really needed? | This table has been deleted and the brief information it contains described as text. |
| Don’t describe sensitivity analysis methods in the results. These should be in the methods and it should be clear if these were pre-specified or post-hoc. | Sensitivity analyses (inclusion of all 6-month data regardless of timing) were pre-specified in the protocol (Section 12.3) to assess attrition impact. Description of the sensitivity analysis has been moved to the method section |
| Reviewer #3: The study is a high quality clinical trial, which is well designed, ethically sound and focuses on addressing the crucial component of ulcer management in Leprosy. Despite the results being neutral, the study contributes valuable evidence and demonstrates high research quality in the NTD settings. | We thank the reviewer for this generous assessment and for highlighting the study's contribution. |
